# Association of Arachidonic Acid–Derived Lipid Mediators With Disease Severity in Patients With Relapsing and Progressive Multiple Sclerosis

**DOI:** 10.1212/WNL.0000000000207459

**Published:** 2023-08-01

**Authors:** Jelle Y. Broos, Floor C. Loonstra, Lodewijk R.J. de Ruiter, Mariam Gouda, Wing Hee Fung, Menno M. Schoonheim, Marieke Heijink, Eva M.M. Strijbis, Charlotte Teunissen, Joep Killestein, Helga E. de Vries, Martin Giera, Bernard M.J. Uitdehaag, Gijs Kooij

**Affiliations:** From the MS Center Amsterdam (J.Y.B., W.H.F., H.E.d.V., G.K.), Molecular Cell Biology and Immunology, Vrije Universiteit Amsterdam, Amsterdam Neuroscience, Amsterdam UMC, location VUmc; Leiden University Medical Centre (LUMC) (J.Y.B., M.H., M.A.G.), Center of Proteomics and Metabolomics; MS Center Amsterdam (F.C.L., L.R.J.d.R., W.H.F., E.M.M.S., J.K., B.M.J.U.), Neurology, Vrije Universiteit Amsterdam, MS Center Amsterdam (M.M.T.E.E.G., C.T.), Neurochemistry Laboratory, Department of Clinical Chemistry, and MS Center Amsterdam (M.M.S.), Anatomy and Neurosciences, Vrije Universiteit Amsterdam, Amsterdam Neuroscience, Amsterdam UMC, location VUmc, the Netherlands.

## Abstract

**Background and Objectives:**

Excessive activation of certain lipid mediator (LM) pathways plays a role in the complex pathogenesis of multiple sclerosis (MS). However, the relationship between bioactive LMs and different aspects of CNS-related pathophysiologic processes remains largely unknown. Therefore, in this study, we assessed the association of bioactive LMs belonging to the ω-3/ω-6 lipid classes with clinical and biochemical (serum neurofilament light [sNfL] and serum glial fibrillary acidic protein [sGFAP]) parameters and MRI-based brain volumes in patients with MS (PwMS) and healthy controls (HCs).

**Methods:**

A targeted high-performance liquid chromatography-tandem mass spectrometry approach was used on plasma samples of PwMS and HCs of the Project Y cohort, a cross-sectional population-based cohort that contains PwMS all born in 1966 in the Netherlands and age-matched HCs. LMs were compared between PwMS and HCs and were correlated with levels of sNfL, sGFAP, disability (Expanded Disability Status Scale [EDSS]), and brain volumes. Finally, significant correlates were included in a backward multivariate regression model to identify which LMs best related to disability.

**Results:**

The study sample consisted of 170 patients with relapsing remitting MS (RRMS), 115 patients with progressive MS (PMS), and 125 HCs. LM profiles of patients with PMS significantly differed from those of patients with RRMS and HCs, particularly patients with PMS showed elevated levels of several arachidonic acid (AA) derivatives. In particular, 15-hydroxyeicosatetraenoic acid (HETE) (*r* = 0.24, *p* < 0.001) correlated (average *r* = 0.2, *p* < 0.05) with clinical and biochemical parameters such as EDSS and sNfL. In addition, higher 15-HETE levels were related to lower total brain (*r* = −0.24, *p* = 0.04) and deep gray matter volumes (*r* = −0.27, *p* = 0.02) in patients with PMS and higher lesion volume (*r* = 0.15, *p* = 0.03) in all PwMS.

**Discussion:**

In PwMS of the same birth year, we show that ω-3 and ω-6 LMs are associated with disability, biochemical parameters (sNfL, GFAP), and MRI measures. Furthermore, our findings indicate that, particularly, in patients with PMS, elevated levels of specific products of the AA pathway, such as 15-HETE, associate with neurodegenerative processes. Our findings highlight the potential relevance of ω-6 LMs in the pathogenesis of MS.

Fatty acid–derived lipid mediators (LMs) play a fundamental role in inflammatory and immune responses because they are able to modulate innate and adaptive immune cells.^1^ Given the central role of LMs in induction, resolution, and chronicity of immune responses, LMs are implicated in several inflammatory disorders.^1,2^ Toward personalized treatment, assessing LMs can provide valuable clues for explaining phenotype variability and possible underlying pathophysiologic mechanisms.

Recent evidence suggests that excessive activation of certain LM pathways, for instance, the arachidonic acid (AA) pathway, plays a role in the complex pathogenesis of multiple sclerosis (MS).^3^ LMs, such as the AA derivative 15-hydroxyeicosatetraenoic acid (HETE) and the linoleic acid (LA) derivative 13-hydroxyoctadecadienoic acid (HODE), have been appreciated for their involvement in inflammatory responses. Both LMs promote lipid/debris uptake through the nuclear peroxisome proliferator–activated receptor gamma (PPARy) in macrophages, thereby enabling tissue repair.^4^ Other key players in LM pathways and pivotal initiators of inflammatory responses are prostaglandins and leukotrienes (eFigure 1, links.lww.com/WNL/C884). We have previously shown that bioactive LM plasma levels are altered in different MS subtypes (e.g., patients with relapsing remitting MS [RRMS] during relapses or in remission and patients with progressive MS [PMS]) when compared with those in healthy controls (HCs).^5^ These findings suggest that altered bioactive LM levels may be associated with impaired resolution responses, thereby potentially contributing to the (chronic) neuroinflammatory process as seen in MS.^6,7^

Despite emerging interest in the role of LMs in MS pathogenesis, the association between LMs and established (bio)markers of neuroinflammation and neurodegeneration remains elusive. An earlier study, consisting of 46 patients with MS (PwMS) and their siblings, could not detect correlations of prostaglandin E_2_ (PGE_2_) and 15(*S*)-HETE with neurofilament light (NfL) and glial fibrillary acidic protein (GFAP),^8^ markers of neuroaxonal damage and astrocyte activation, respectively. Inflammation-activated astrocytes have been shown to increase the production and secretion of polyunsaturated fatty acids (PUFAs) in the brain, and GFAP is therefore expected to show relationships with LMs.^9,10^ Absent correlations of the aforementioned study, however, might be due to the study design and warrant further exploration, also including other derivatives of ω-3 and ω-6 LM pathways. Furthermore, the relationship between LMs and MRI-derived brain volumes, which are known to strongly relate to clinical progression and PMS,^11^ remains unknown.

To better understand the relationship between bioactive LMs and different aspects of CNS related pathophysiologic processes in MS, in this study, we investigated bioactive LM levels belonging to the ω-3/ω-6 lipid classes in plasma of different MS phenotypes, and we determined whether and how these levels relate to disability outcome measures, brain volumes, and the blood biomarkers serum NfL (sNfL) and serum GFAP (sGFAP). LM levels were determined by high-performance liquid chromatography-tandem mass spectrometry (HPLC-MS/MS) on plasma samples from a large population-based cohort of PwMS all born in 1966 and age-matched HCs (Project Y).^12^ By using a cohort of PwMS and HCs of the same age, we have limited the confounding effect of age because age is known to affect LM concentrations.^13,14^

## Methods

### Study Population

PwMS and HCs were selected from the cohort study Project Y. Project Y is a population-based cross-sectional birth year cohort, which aimed to include all PwMS (as defined by the 2017 McDonald criteria)^15^ born in 1966 in the Netherlands and age-matched and sex-matched HCs born in between 1965 and 1967 in the Netherlands. Potential participants were excluded if they were unable to undergo minimal data collection and/or were not currently living in the Netherlands. Patients diagnosed with MS before study participation and HCs were subjected to comprehensive examinations during a 1-day study visit (single time point) between December 2017 and January 2021 at the Amsterdam University Medical Center (Amsterdam UMC), location VUmc. Details of the study design and total study population have been previously described.^12^

### Standard Protocol Approvals, Registrations, and Patient Consents

The Project Y protocol was approved by the Medical Ethical Committee of the Amsterdam UMC, location VUmc, and all participants gave written informed consent before participation.

### Targeted LM Analysis

Blood samples were collected through standard venipuncture during the study visit and were stored at −80°C until further analysis. Plasma samples (400 μL) were quenched by adding 1.2 mL of methanol (MeOH) and 4 µL of internal standard solution consisting of leukotriene B_4_-d4 (LTB_4_-d4), 15-HETE-d8, PGE_2_-d4, docosahexaenoic acid (DHA)-d5, and 9(S)-HODE-d4 (50 ng/mL in MeOH). In addition, 40 μL of every sample was pooled and used as quality control once every 10 samples to ensure the quality of the experiment. Samples were placed at −20°C for 20 minutes and spun down for 10 minutes at 16,200*g* at 4°C. Supernatants were then diluted in 6 mL of H_2_O, and pH was corrected to 3.5 using formic acid (99%) after which solid phase extraction (SPE) was used to further isolate the LMs. For SPE, C-18 cartridges (Sep-Pak, Vac3 3 mL [200 mg]) were used, prewashed with MeOH and H_2_O. After sample loading, the C-18 cartridges were washed with H_2_O and n-hexane after which the extracted LMs were eluted using methylformate and collected in glass tubes. The elutes were dried at 40°C for 1 hour using an N2 flow, reconstructed in 100 µL of MeOH (40%) and transferred to deactivated glass inserts. Ω-3/Ω-6 LM content of the samples was measured using a targeted HPLC-MS/MS method.^16,17^ LMs were detected using their relative retention times together with characteristic mass transitions, and these and other individually optimized parameters are summarized in eTable 1 (links.lww.com/WNL/C884).

Before statistical analyses, all LM data were assessed first, and LMs were excluded from the dataset if missing values were found in >150 samples. Hereafter, the still remaining missing values were imputed with 1/5 of the lowest value for each LM, and the data were then normalized using total area normalization to obtain relative values as previously described.^18^ In total, 39 LMs were used for statistical analyses, which were performed in RStudio (version 1.4.1106).

### sNfL and GFAP

sNfL and sGFAP were measured using a single-molecule array assay on a HD-X analyzer, according to the manufacturer's instructions (Quanterix, Billerica, MA). Analyses were performed at the Neurochemistry laboratory of the Department of Clinical Chemistry (Amsterdam UMC, location VUmc). sNfL and sGFAP measurements are described in detail elsewhere.^19^ Quality controls showed an intra-assay coefficient of variation of sGFAP below the accepted threshold of <20%.^20^

### Imaging

All participants were scanned on a 3T whole-body MRI scanner; the MRI protocol has been previously described.^12^ In brief, the protocol included 3-dimensional (3D) T1-weighted images (1 mm isotropic) for volumetric measurements (using FMRIB Software Library) and a 3D fluid-attenuated inversion recovery sequence (also 1 mm isotropic) for white matter lesion segmentation. Normalized total brain volume (NTBV) and normal white matter volume (NWMV) were calculated on lesion-filled T1 images using SIENAX. FAST (part of SIENAX) and FIRST were used to segment total gray matter volume and normalized deep gray matter volume (NDGMV) also including thalamic volume; normalized cortical gray matter volume (NCGMV) was obtained by subtracting deep gray matter segmentations from total gray matter segmentations using FSLmaths. All volumetric measures, except lesion volumes (LVs), were normalized for head size using SIENAX-derived V scaling.

### Clinical Assessment

All participants were subjected to comprehensive examinations during a 1-day study visit, including a detailed interview regarding (MS) disease history. PwMS were asked about the date of onset and diagnosis, MS phenotype, use of disease-modifying therapy (DMT), and general medical history. The patient's reported medical history was corroborated by medical records. Overall MS-related physical disability was measured using the Expanded Disability Status Scale (EDSS).

### Data Handling and Statistical Analysis

Statistical analysis was performed using RStudio (version 1.4.1106). Normality of the data was checked by visual inspection of histograms combined with Kolmogorov-Smirnov testing. A *p* value ≤0.05 was considered as statistically significant. Because our research is hypothesis generating, data were unadjusted for multiple testing; however, false discovery rate (FDR)–corrected data are added in supplementary tables (eTables 1 and 2, links.lww.com/WNL/C884). Both primary and secondary progressive PwMS were grouped as PMS to increase statistical power. Our statistical analysis consisted of 4 stages.

#### Partial Least Squares Discriminant Analysis

First, we performed a partial least squares discriminant analysis (PLS-DA) using the mixOmics package in R. For this, we included all 39 LMs to optimize the separation of our 3 cohort groups (eFigure 2A, links.lww.com/WNL/C884) and to identify which LMs contributed most to this separation as indicated by the variable importance in projection (VIP) score (eFigure 2B). Based on this, we selected only those 14 LMs with a VIP score >1 for further analyses because these LMs were believed to be the most contributory variables for cohort group discrimination.

#### Cluster Analysis and Group Comparisons

Hereafter, we performed a 2-way hierarchical cluster analysis using the “ComplexHeatmap” package in R.^21^ Three sample cluster (SC) groups, matching the 3 cohort groups and 2 LM groups, matching the 2 used LM pathways, were given to the algorithm as input, and data were clustered using the Euclidean distance method of the R package. A χ^2^ test was performed on the quantitative output of the 3 SCs. Second, mean LM levels were compared between all subgroups (HCs; patients with RRMS; and patients with PMS) using volcano plots where the log2 fold change was plotted against the –log10 *p* value. Group comparisons of individual LM's were performed using nonparametric tests (Mann-Whitney *U* test). Moreover, LM levels were compared between PwMS using first-line DMT (dimethyl fumarate, interferon, teriflunomide, and glatiramer acetate) and second-line DMT (natalizumab, ocrelizumab, and fingolimod; data not shown).

#### Correlation Analyses

Next, non-normally distributed parameters (e.g., sNFL, GFAP, LV, and all LMs values) were transformed using a Box-Cox transformation and used in partial correlation analyses together with the EDSS and other MRI volumetric measures. As initial screening approach and for variable reduction, the relationship of LMs with disability (EDSS), sNfL, GFAP, and MRI volumetric measures was analyzed using Pearson correlations in which sex, disease duration, smoking, body mass index (BMI), and DMT use during sampling (present/absent) were used as covariates.

#### Linear Regression Analysis

To further reduce the number of tests for LM analyses, significant correlates of the EDSS (5-HETE, 8-HETE, 15-HETE, dihomo-γ-linolenic acid [DGLA] and adrenic acid [AdA]) were fed into a multivariate linear regression model using a backward selection procedure with a removal *p* value of >0.10. BMI, DMT use (present vs absent), disease duration, MS subtype (progressive vs relapsing), smoking (ever vs never), and sex (female vs male) were included as covariates. The outcome of this model identified LMs that best related to disability (as indicated with the EDSS). Finally, to assess whether LMs, sNfL, and sGFAP were independently related to disability, the aforementioned final model was repeated forcing sNfL and sGFAP in the final model.

### Data Availability

Anonymized data, not published in the article, will be shared on reasonable request from a qualified investigator.

## Results

### Patient Characteristics

Of the 367 PwMS and 125 HCs who participated in the Project Y cohort, a total of 285 PwMS (170 patients with RRMS and 115 patients with PMS) and 125 age-matched HCs had available serum samples and were selected for the aim of this study. The main characteristics of the study populations are summarized in Table 1. In short, we observed higher median EDSS scores in patients with PMS (EDSS = 5.5, interquartile range [IQR] 4.0–6.5) vs those in patients with RRMS (EDSS = 3.0, IQR 2.0–4.0) (*p* < 0.001). Moreover, sNfL and sGFAP levels were significantly higher in PwMS than in HCs. When comparing subgroups, patients with PMS had significantly higher sNfL and sGFAP levels compared with patients with RRMS. All brain volumes were lower in PwMS vs those in HCs (all *p* < 0.001). In particular, all gray matter volumes were lower in patients with PMS vs patients with RRMS.

**Table 1:**
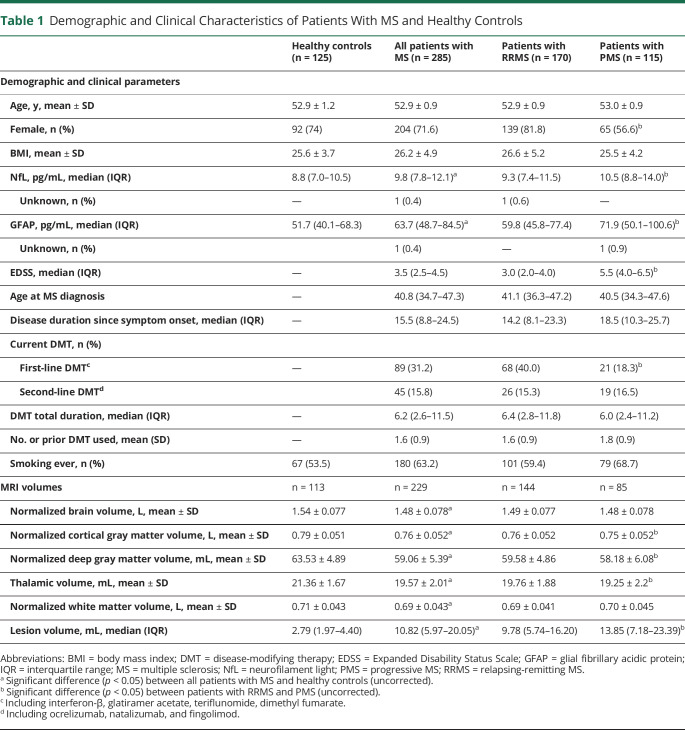
Demographic and Clinical Characteristics of Patients With MS and Healthy Controls

### Hierarchical Clustering Shows LM Profile Differences Between HCs and Those With PMS

**Figure 1:**
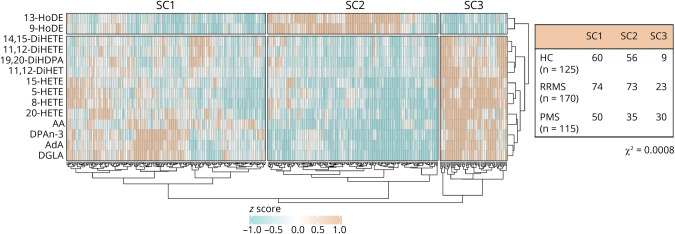
Two-Way Hierarchical Clustered Heatmap Shows Differences in LM Profiles Related to the ω-3/ω-6 Lipid Pathways Heatmap with hierarchical cluster analysis (Euclidean distance) showing the distribution of Project Y cohort participants (x-axis) over the 3 sample clusters that are based on the ω-3/ω-6 lipid mediator plasma levels (y-axis). Table indicates the cohort group distribution over the sample clusters (χ^2^ test = 0.0008). AA = arachidonic acid; AdA = adrenic acid; DGLA = dihomo-γ-linolenic acid; DHA = docosahexaenoic acid; DiHDPA = dihydroxydocosapentaenoic acid; DiHET = dihydroxyeicosatrienoic acid; DiHETE = dihydroxyeicosatetraenoic acid; DPA = docosapentaenoic acid; HETE = hydroxyeicosatetraenoic acid; HODE = hydroxyoctadecadienoic acid; LM = lipid mediator; HC = healthy control; MS = multiple sclerosis; PMS = progressive MS; RRMS = relapsing-remitting MS; SC = sample cluster.

To determine whether PwMS show altered ω-3/ω-6 LM plasma levels, a PLS-DA was first performed to assess and optimize the separation of the 3 subgroups (HCs, people with RRMS, and people with PMS) (eFigure 2, A and B, links.lww.com/WNL/C884). Fourteen LMs (VIP score >1) contributed to this separation; therefore, only these LMs were used for subsequent analyses. A 2-way hierarchical clustering method resulted in 3 SCs (vertical) and 2 LM clusters (horizontal, Figure 1). The horizontal LM clusters were formed based on their origin in the ω-3/ω-6 pathways (eFigure 3, A and B); 1 LM cluster consisted of derivatives of the upstream PUFA LA (e.g., HODE), while the other LM cluster consisted of LMs associated with the downstream PUFAs AA, eicosapentaenoic acid (EPA), and DHA (e.g., HETEs, dihydroxyeicosatetraenoic acids [DiHETEs] and 19,20-dihydroxydocosapentaenoic acid [DiHDPA]). The 3 vertical SCs were formed based on the individual LM profiles. SC1 contained participants with low to average levels of the HODE and average to high levels of the other LMs. SC2 displayed relatively high levels of both 9-HODE and 13-HODE and relatively low to average levels of DGLA, AA, HETEs, DiHETEs, and 19,20-DiHDPA. SC3 showed low levels of the HODEs and hydroxyoctadecatrienoic acid but average to high levels of all the other LMs. After this initial clustering, we determined the distribution of the 3 subgroups (HCs, people with RRMS, and people with PMS) over these 3 SCs using a χ^2^ test (Figure 1). The distribution was different than what could be expected based on the number of participants per subgroup (χ^2^ < 0.0008). SC3 seems to be mainly responsible for this because this cluster contains relatively few HCs and patients with RRMS and a relatively large amount of patients with PMS.

### Mean LM Levels Show a Distinct LM Profile in Patients With PMS

**Figure 2:**
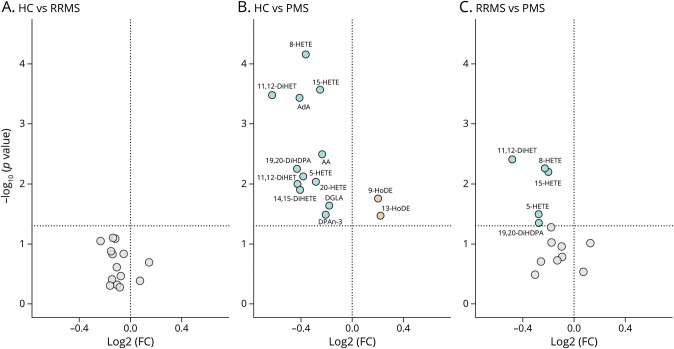
Volcano Plots Displaying Significant Differences of ω-3/ω-6 LMs Between HC and MS Groups Volcano plots showing the log2 fold change of LMs between the HCs and (A) patients with RRMS and (B) patients with PMS. (C) Log2 fold change of LMs between patients with RRMS and PMS. Level of significance is indicated on the y-axis as –log10(*p* value). AA = arachidonic acid; AdA = adrenic acid; DGLA = dihomo-γ-linolenic acid; DHA = docosahexaenoic acid; DiHDPA = dihydroxydocosapentaenoic acid; DiHET = dihydroxyeicosatrienoic acid; DiHETE = dihydroxyeicosatetraenoic acid; DPA = docosapentaenoic acid; HETE = hydroxyeicosatetraenoic acid; HODE = hydroxyoctadecadienoic acid; LM = lipid mediator; HC = healthy control; MS = multiple sclerosis; PMS = progressive MS; RRMS = relapsing-remitting MS.

To confirm that the PMS lipid profile indeed differed from that of HCs and patients with RRMS, mean LM levels were compared between these subgroups (Figure 2, A–C). Significant differences in LM levels were seen between patients with PMS and RRMS and between patients with PMS and HCs (Figure 2, B and C), whereas no significant differences were observed between patients with RRMS and HCs (Figure 2A). Twelve LMs, predominantly associated with AA (e.g., 5-HETE, 8-HETE, and 15-HETE), were significantly higher in the PMS group compared with those in HCs, while 2 LMs, 9-HODE and 13-HODE, associated with the more upstream LA, were significantly decreased in patients with PMS compared with that in HCs (Figure 2B). Of importance, all the observed differences in LM levels between patients with PMS and HCs survived FDR correction (eTable 2, links.lww.com/WNL/C884). Five of these 12 significantly elevated LMs were also significantly higher in the PMS group compared with that in patients with RRMS (Figure 2C), although part of this significance was lost after FDR correction (eTable 2). Furthermore, no significant changes in LM levels were found between PwMS using first-line DMT and those using second-line DMT. Taken together, we demonstrate, in this study, an altered LM profile in patients with PMS compared with that in patients with RRMS and HCs, which is predominantly linked to the ω-6 lipid pathway. In addition, this altered LM profile is characterized by increased levels of AA derivatives such as 8-HETE and 15-HETE and decreased levels of LA derivatives such as 9-HODE and 13-HODE.

### Ω-6 and ω-3 LM–Derived LMs Correlate With Disability, sNfL, and sGFAP

**Figure 3:**
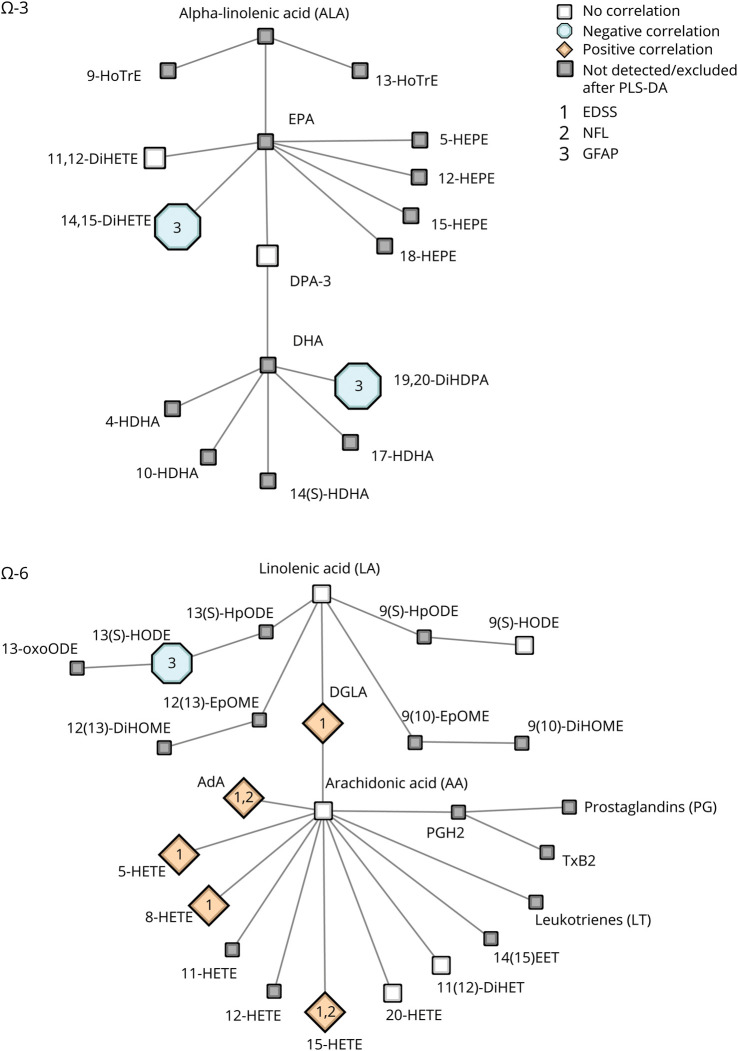
Schematic Overview of ω-3/ω-6 LM Pathways and Correlations With Clinical Parameters This figure represents all significant correlations between lipid mediators, clinical and biochemical parameters (EDSS, NfL, and GFAP) in both patients with progressive MS and in the whole MS group. Symbols and colors are indicative of the direction of the correlation (orange/hexagonal = positive correlation, blue/square = negative correlation), and numbers are indicative for the clinical/biochemical correlate (1 = EDSS, 2 = sNFL, 3 = sGFAP). AdA = adrenic acid; DGLA = dihomo-γ-linolenic acid; DHA = docosahexaenoic acid; DiHDPA = dihydroxydocosapentaenoic acid; DiHET = dihydroxyeicosatrienoic acid; DiHETE = dihydroxyeicosatetraenoic acid; DPA = docosapentaenoic acid; EDSS = Expanded Disability Status Scale; EPA = eicosapentaenoic acid; HETE = hydroxyeicosatetraenoic acid; HODE = hydroxyoctadecadienoic acid; LA = linoleic acid; LM = lipid mediator; GFAP = glial fibrillary acidic protein; LM = lipid mediator; NfL = neurofilament light; PLS-DA = partial least squares discriminant analysis; sGFAP = serum GFAP; sNfL = serum NfL.

To assess whether the observed LM differences associate with clinical measures and pathologic processes such as axonal damage and astrocyte reactivity, LM levels were correlated with the EDSS, sNfL, and sGFAP. Both positive and negative correlations were observed between clinical parameters and ω-6–associated LMs (Table 2 and Figure 3). In the PMS group, higher levels of the AA derivatives 8-HETE and 15-HETE correlated with worse EDSS scores. Moreover, in patients with PMS, high sNfL levels were related to elevated 15-HETE levels. The LA derivative 13-HODE negatively correlated with sGFAP in both the RRMS group and in the whole MS group (Table 2 and Figure 3). In the whole MS group, similar to patients with PMS, worse EDSS scores and higher sNfL levels correlated not only with increased levels of several AA-derived HETEs (e.g., 5-HETE, 8-HETE, and15-HETE) but also with increased levels of the PUFAs DGLA and AdA.

**Table 2:**
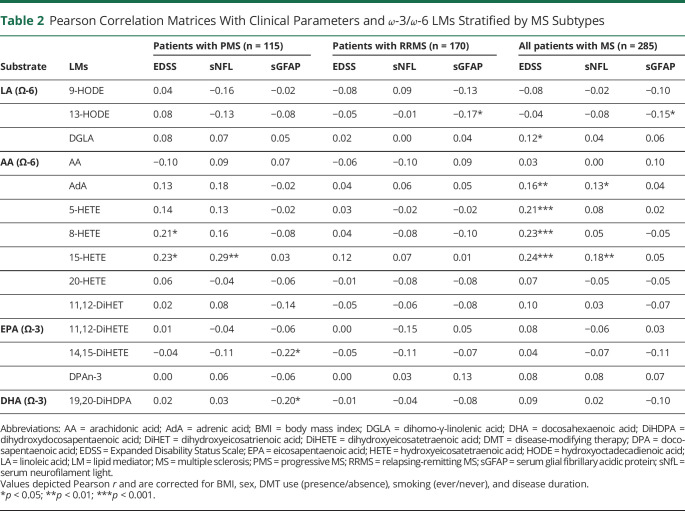
Pearson Correlation Matrices With Clinical Parameters and ω-3/ω-6 LMs Stratified by MS Subtypes

Correlations with clinical parameters and ω-3 LMs were also assessed (Table 2 and Figure 3). In this study, only negative correlations with sGFAP levels and the EPA derivative 14,15-DiHETE and DHA derivative 19,20-DiHDPA were found in patients with PMS. No correlations were observed in patients with RRMS and in the whole MS group. In summary, these findings show that EDSS and sNFL seem to be predominantly associated with AA derivatives, whereas sGFAP levels may be more associated with both LA derivatives and ω-3 associated LMs.

### Ω-6 and ω-3 LMs Correlate With MRI-Derived Volumetric Measures

To further assess the potential link between LMs and neurodegenerative processes, we next correlated LM levels with brain volumes (Table 3). In general, we observed that in patients with PMS, increased levels of the AA derivative 15-HETE were related with worse atrophic changes. Specifically, higher levels of 15-HETE correlated with both lower NTBV and NDGMV in this subgroup. Higher levels of the ω-6 PUFA AdA correlated with higher LV. Furthermore, lower levels of both EPA derivatives 11,12-DiHETE and 14,15-DiHETE correlated with lower thalamic volume in patients with PMS.

**Table 3:**
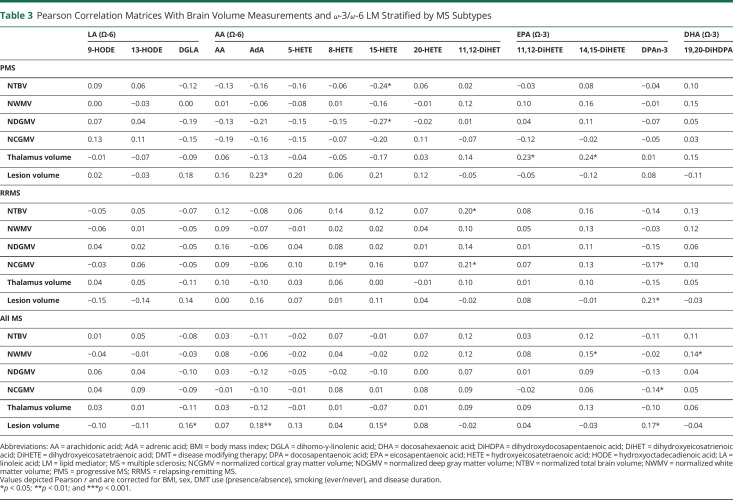
Pearson Correlation Matrices With Brain Volume Measurements and ω-3/ω-6 LM Stratified by MS Subtypes

In the RRMS group, higher levels of the AA derivative 11,12-dihydroxyeicosatrienoic acid (DiHET) were significantly related with higher NBV (Table 3). In addition, both higher 11,12-DiHET levels and higher 8-HETE levels correlated with higher NCGMV, whereas higher ω-3 PUFA docosapentaenoic acid (DPA)n-3 correlated with lower NCGCV. Moreover, in the RRMS group, higher DPAn-3 showed a positive correlation with higher LV. In the whole MS group, higher DGLA, AdA, 15-HETE, and DPAn-3 levels correlated with higher LV. In addition, higher 14,15-DiHETE and 19,20-DiHDPA levels were significantly related to NWMVs in this group. High levels of DPAn-3, on the contrary, correlated with lower NCGMV. Taken together, these findings demonstrate that several ω-6 and ω-3 derivatives, such as AdA, 15-HETE, DPAn-3, 11,12-DiHET, and 8-HETE, relate to LV or brain atrophy as determined by MRI.

### Linear Regression: 15-HETE Best Related to Disability

Finally, to identify which LMs best related to disability (EDSS), a linear regression was performed. The backward selection linear regression model included sex, disease duration, BMI, DMT use, MS subtype, smoking status, and all LMs, which significantly correlated with the EDSS. The final model (Table 4) indicated that worse disability was associated with higher 15-HETE, longer disease duration, and progressive disease (*R*^2^ = 0.456). In addition, the final model was repeated after also adding sNfL and sGFAP as fixed variables, showing significant associations of 15-HETE, disease duration, MS subtype, sNfL, and sGFAP with disability in all PwMS (*R*^2^ = 0.482, *p* < 0.001).

**Table 4:**
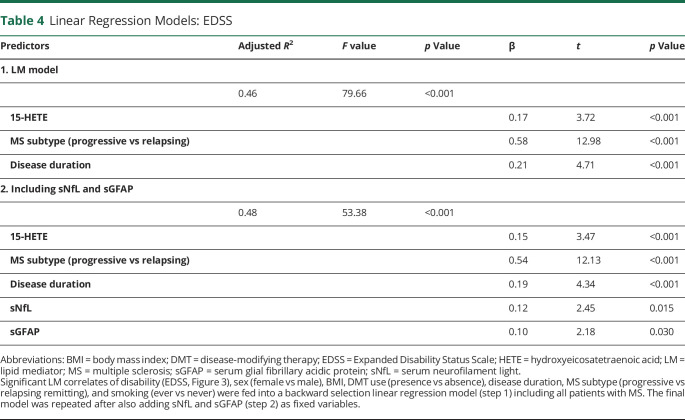
Linear Regression Models: EDSS

## Discussion

In our cohort of PwMS and HCs of the same age, we found that patients with PMS particularly show a different LM profile compared with both patients with RRMS and HC, characterized predominantly by an increase of AA and its derivatives (e.g., 5-HETE, 8-HETE, and 15-HETE). We found several correlations between ω-3/ω-6 LM plasma levels and disability (EDSS), biochemical parameters (sNfL, sGFAP), and MRI measures in both patients with RRMS and PMS. The AA derivative 15-HETE was of particular interest because higher 15-HETE levels were not only related to higher sNfL concentrations and EDSS, but also to lower NTBV and NDGMV in the PMS group. Finally, worse disability was best explained by higher 15-HETE levels and longer disease duration. Taken together, these findings indicate that particularly in patients with PMS, specific parts of the AA pathway are associated with neurodegenerative processes, reflected by disability. Because previous studies on the role of LMs are affected by age, the main strength of this study is the inclusion of patients and HCs of the same age. Age is known to affect LM concentrations^13^ and aging is, among other, associated with a deficiency of bioactive LMs such as specialized pro-resolving mediators.^14^ By using a cohort of PwMS and HCs of the same age, we have limited the confounding effect of age. Besides, analyses were adjusted for sex, given the different results seen in male and female individuals in earlier studies, such as increased 11,12-DHET in the CSF of male PwMS vs female PwMS.^22^ We were able to observe correlations between higher 15-HETE levels, lower NTBV and NDGMV, and higher LV, suggesting a potential link between 15-HETE and CNS-associated pathologic processes in MS. This is further supported by the positive correlations we observed between 15-HETE and both sNfL and disability in PMS, indicating that 15-HETE is associated with neurodegeneration.

To date, no direct correlations between MRI measures and 15-HETE have been described earlier. A possible explanation for the detrimental effects of 15-HETE can be found in its ability to bind to several receptors, including leukotriene B_4_ receptor 2 and PPARy, through which it can regulate several cellular processes including apoptosis.^23,24^ In addition, 15-HETE can display additional detrimental effects because it is able to induce reactive oxygen species production,^25^ which might be involved in neurodegenerative processes.^26^ Moreover, 15-HETE may promote foam cell formation, a process also observed in MS in which CNS infiltrated macrophages become oversaturated with oxidized lipids.^16,17,27^ An earlier study demonstrated that silencing of human arachidonate 15-lipoxygenase type B (ALOX15-B) in human primary macrophages, one of the enzymes responsible for the biosynthesis of the isoform 15(S)-HETE, led to a decrease in lipid accumulation and inflammatory markers.^28^ In addition, by using a knockdown of the mouse Alox15b gene in LDL-receptor deficient (Ldlr (−/−)) mice, it was shown that ALOX15-B is linked to increased foam cell size and plaque lipid content in atherosclerotic plaques.^28^ These findings suggest that ALOX15-B and presumably its LM product 15(S)-HETE are associated with lipid uptake, which is a critical process in MS. Myelin internalization by foamy phagocytes is not only a disease-promoting process by presenting brain-derived autoantigens and adopting an inflammatory phenotype but also a prerequisite for CNS repair because damaged myelin clearance is essential to facilitate remyelination.^27^ However, additional experiments are required to substantiate this hypothesis and to uncover the cellular source and function of 15-HETE in the context of MS. Of note, both 8-HETE and 15-HETE are produced by the enzyme ALOX-15B, which could explain the similar trend observed in their relation with disability.^29^ In addition, the backward selection procedure, which was used to create final linear regression models for disability, might have resulted in the removal of 8-HETE in these models because of the similar effect of 15-HETE and 8-HETE on the variance in EDSS.

In line with our results, previous studies have documented elevated levels of both 15-HETE and 15(S)-HETE in PwMS compared with controls^6,8,30^ and higher 15-HETE CSF levels in patients with Alzheimer disease compared with patients with mild cognitive impairment and subjective impairment.^31^ In our previous work, significantly increased levels of 15-HETE were already found in the plasma of progressive PwMS compared with those in HCs, yet no correlation with clinical data was observed, presumably due to the small sample size.^6^ Moreover, in the Gothenburg MS registry, elevated CSF levels of both 15(S)-HETE and PGE_2_ were found in PwMS compared with those in healthy siblings and controls.^8^ However, no correlations with either EDSS, sNFL, or sGFAP were observed in this study, which might be explained by the small cohort size (n = 46), less advanced measuring techniques, or by the fact that both patients with progressive MS (n = 27) and those with RRMS (n = 19) were grouped together. While we observed significant correlations between 15-HETE and sNfL in PMS and in the whole MS group, there was no evidence of a significant correlation between 15-HETE (or other AA derivatives) and sGFAP in our cohort. Finally, we reported independent associations of 15-HETE, sNfL, and sGFAP with EDSS in all PwMS.

The AA derivative 11,12-DiHET (ω-6) was also identified as an interesting target in our cohort because higher levels of this LM correlated with higher NBV and NCGMV. An earlier study reported increased levels of 11,12-DiHET in relapsing PwMS compared with nonrelapsing PwMS.^22^ Originally, DiHETs are believed to be nonfunctional degradation products of the EETs,^32^ which possess anti-inflammatory and neuroprotective properties under pathophysiologic circumstances such as cerebral ischemia.^33,34^ The positive correlation between 11,12-DiHET and brain volumes in our study could therefore be the result of higher levels of the functional but relatively unstable precursor 11,12-EET. However, some studies have proposed that several DiHETs, including 11,12-DiHET, may have some biological functions similar to their EET precursor, and therefore, 11,12-DiHET itself may be crucial in protecting the CNS against cellular damage.^29^ However, these findings warrant further exploration.

In our cohort, plasma levels of the LA derivatives 9-HODE and 13-HODE were significantly lower in PMS compared with those in HCs. This decrease in LA derivatives in PMS, together with elevated levels of the more downstream AA derivatives, could be indicative of an LM switch in the ω-6 lipid pathway. We therefore speculate that changes in enzyme expression and/or activity required for the synthesis of ω-3/ω-6 PUFA derivatives (e.g., ALOX-5,15-1/B, cyclooxygenase-1/2) may result in higher levels of 15-HETE and lower levels of 13-HODE, presumably due to higher expression of ALOX15-B.^35^ This is based on the fact that ALOX15-1, the main producer of 13-HODE, more efficiently metabolizes LA than AA, whereas ALOX15-B, the main producer of 15-HETE, metabolizes LA very poorly but in turn very efficiently metabolizes AA.^36^ Decreased 13-HODE levels were also (weakly) related to increased sGFAP levels in patients with RRMS and in the whole MS group. In the CNS, astrocytes are a major source of fatty acid synthesis, and we therefore speculate that astrocyte activation is linked to changes in the LM profile of PwMS.^9,10^ However, additional studies are necessary to further assess this hypothesis.

In contrast to other studies, proinflammatory LMs such as PGE_2_ and LTB_4_ and LTD_4_, levels of which are generally elevated and linked to severe inflammation,^5^ were insufficiently detectable in our study to include them in our data analysis. This limitation has been previously described^22^ and may arise from the fact that PGE_2_ has a rapid turnover rate in vivo.^37^ Another possible explanation could be the relatively high age of our cohort combined with a long disease duration and its associated decrease of inflammatory activity compared with younger equivalents, thus showing reduced levels of these proinflammatory LMs. We therefore speculate that the correlations between other AA derivatives and our clinical, biochemical, and MRI parameters do not reflect acute inflammation per se but may represent the neurodegenerative processes in the CNS.

Several limitations of this study need to be addressed. Due to the cross-sectional nature of this study, conclusions about causality cannot be made. Furthermore, because our study design did not include gadolinium administration, we were not able to differentiate between active and inactive inflammatory CNS lesions. Next, we assessed only the EDSS as clinical measure. Although the EDSS is the most frequent used outcome measure in MS, the relationship between LMs and other clinical (disability) measures might have revealed more detailed information. Based on our results, LMs could be useful in clinical trials addressing PMS at group level; however, considering the overlap between patients with PMS and RRMS, LMs might be less valuable to inform clinical decisions at the individual level. A possible explanation for this overlap could be the higher age of the patients and the associated decrease in inflammation. Moreover, because the observed correlations between LMs and study outcome measures were weak, our results need confirmation by replication studies. Large longitudinal studies are warranted to study plasma and CSF LM profiles in PwMS with acute inflammation, during remission and during the more progressive phase of the disease, to define the exact LM profiles during disease pathogenesis and their potential contribution to neuropathologic events in MS. These studies should control for all DMT types because we considered DMT as present or absent.

In conclusion, in a cohort of patients of the same age, we show that several of the ω-3/ω-6 associated LMs are related to CNS-related pathophysiologic processes in MS. We demonstrate that patients with PMS particularly display an altered LM profile compared with those with RRMS and HCs. This altered LM profile in PMS is mainly driven by increased levels of AA and its derivatives, which correlated to volumetric MRI measures, sNfL, and disability. The AA derivative 15-HETE best related to the EDSS, indicating that this AA derivative may mark neurodegeneration, reflected by disability. In general, these findings highlight the potential relevance of LMs in the pathogenesis of MS.

## Glossary

3D: 3-dimensional
AA: arachidonic acid
AdA: adrenic acid
ALOX15-B: arachidonate 15-lipoxygenase type B
Amsterdam UMC: Amsterdam University Medical Center
BMI: body mass index
DGLA: dihomo-γ-linolenic acid
DHA: docosahexaenoic acid
DiHDPA: dihydroxydocosapentaenoic acid
DiHET: dihydroxyeicosatrienoic acid
DiHETE: dihydroxyeicosatetraenoic acid
DMT: disease-modifying therapy
DPA: docosapentaenoic acid
EDSS: Expanded Disability Status Scale
EPA: eicosapentaenoic acid
FDR: false discovery rate
GFAP: glial fibrillary acidic protein
HC: healthy control
HETE: hydroxyeicosatetraenoic acid
HODE: hydroxyoctadecadienoic acid
HPLC-MS/MS: high-performance liquid chromatography-tandem mass spectrometry
IQR: interquartile range
LA: linoleic acid
LM: lipid mediator
LTB_4_-d4: leukotriene B_4_-d4
LV: lesion volume
MeOH: methanol
MS: multiple sclerosis
NCGMV: normalized cortical gray matter volume
NDGMV: normalized deep gray matter volume
NTBV: normalized total brain volume
NWMV: normalized white matter volume
NfL: neurofilament light
PGE_2_: prostaglandin E_2_
PLS-DA: partial least squares discriminant analysis
PMS: progressive MS
PPARy: peroxisome proliferator–activated receptor gamma
PUFA: polyunsaturated fatty acid
PwMS: patients with MS
RRMS: relapsing-remitting MS
SC: sample cluster
sGFAP: serum GFAP
sNfL: serum NfL
SPE: solid phase extraction
VIP: variable importance in projection
